# The non-invasive exfoliated transcriptome (exfoliome) reflects the tissue-level transcriptome in a mouse model of NSAID enteropathy

**DOI:** 10.1038/s41598-017-13999-5

**Published:** 2017-10-31

**Authors:** Canaan M. Whitfield-Cargile, Noah D. Cohen, Kejun He, Ivan Ivanov, Jennifer S. Goldsby, Ana Chamoun-Emanuelli, Brad R. Weeks, Laurie A. Davidson, Robert S. Chapkin

**Affiliations:** 1 Department of Large Animal Clinical Sciences, College of Veterinary Medicine & Biomedical Sciences, Texas A&M University, College Station, Texas, United States of America; 2 Department of Statistics, College of Science, Texas A&M University, College Station, Texas, USA; 3 Department of Veterinary Physiology and Pharmacology, College of Veterinary Medicine & Biomedical Sciences, Texas A&M University, College Station, Texas, USA; 4 Program in Integrative Nutrition & Complex Diseases, College of Agriculture and Life Sciences, Texas A&M University, College Station, Texas, USA; 5 Center for Translational Environmental Health Research, Texas A&M University, College Station, Texas, USA; 6 Department of Veterinary Pathobiology, College of Veterinary Medicine & Biomedical Sciences, Texas A&M University, College Station, Texas, USA; 7 Present Address: Institute of Statistics and Big Data, Renmin University of China, Beijing, 100872 China

## Abstract

Non-steroidal anti-inflammatory drugs (NSAIDs) are among the most frequently used classes of medications in the world, yet they induce an enteropathy that is associated with high morbidity and mortality. A major limitation to better understanding the pathophysiology and diagnosis of this enteropathy is the difficulty of obtaining information about the primary site of injury, namely the distal small intestine. We investigated the utility of using mRNA from exfoliated cells in stool as a means to surveil the distal small intestine in a murine model of NSAID enteropathy. Specifically, we performed RNA-Seq on exfoliated cells found in feces and compared these data to RNA-Seq from both the small intestinal mucosa and colonic mucosa of healthy control mice or those exhibiting NSAID-induced enteropathy. Global gene expression analysis, data intersection, pathway analysis, and computational approaches including linear discriminant analysis (LDA) and sparse canonical correlation analysis (CCA) were used to assess the inter-relatedness of tissue (invasive) and stool (noninvasive) datasets. These analyses revealed that the exfoliated cell transcriptome closely mirrored the transcriptome of the small intestinal mucosa. Thus, the exfoliome may serve as a non-invasive means of detecting and monitoring NSAID enteropathy (and possibly other gastrointestinal mucosal inflammatory diseases).

## Introduction

Non-steroidal anti-inflammatory drugs (NSAIDs) are among the most frequently consumed pharmaceuticals worldwide because of their anti-inflammatory, anti-neoplastic, and analgesic effects. Their use can result in an enteropathy that has an alarmingly high rate of morbidity and mortality. In the United States alone, NSAID enteropathy results in approximately 100,000 hospitalizations and 16,500 deaths each year^1^. An additional 2/3 of both short- and long-term NSAID users develop subclinical or undiagnosed distal small intestinal lesions^2^. Although detection and management of NSAID-induced lesions of the *proximal* GI tract (*i*.*e*., gastropathy) are well documented, diagnosis and treatment of NSAID-induced damage to the GI tract *distal to the duodenum* (also known as NSAID enteropathy, affecting primarily the distal jejunum and ileum) remain elusive^3,4^. This is noteworthy because the incidence of NSAID enteropathy is expected to increase as a result of greater use of NSAIDs to treat rising numbers of inflammatory conditions, to meet the needs of aging populations in North America and Europe, and for their anti-neoplastic effects^5^. The lower GI tract of multiple mammalian species is affected by NSAIDs in a similar manner in terms of anatomic location, pathological findings, and severity of clinical signs^6–8^.

The pathophysiology of NSAID enteropathy is complex and poorly understood^9^. Deleterious effects of NSAIDs on the intestinal mucosa including enterocyte cell death, increased mucosal permeability, and interaction of the damaged mucosa with luminal contents including bacteria (*i*.*e*., GI microbiota) and bacterial products or components such as lipopolysaccharide (LPS)^4,10^ has been proposed. The resulting inflammatory cascade is mediated by the innate immune response to LPS and several pro-inflammatory cytokines including tumor necrosis factor (TNF), interleukin (IL)-1, and IL-6^11–13^. Although the GI microbiota has recently been implicated as an important contributor to NSAID enteropathy, the precise mechanisms of host-microbiota interactions remain to be elucidated^14–18^.

An important limitation to understanding the pathogenesis of NSAID enteropathy is the difficulty in obtaining longitudinal (sequential) data from individuals regarding intestinal function and health. A great clinical and investigative need exists to develop non-invasive methods to characterize the health and function of the GI tract distal to the stomach to more effectively identify, study, and manage NSAID enteropathy. A potential strategy to address this limitation is the use of exfoliated intestinal epithelial cells (IECs) and other cell types found in voided stool. Approximately 1/3 of human colonic epithelial cells (up to 10^10^ cells in an adult) are exfoliated and shed in feces each day^19^. Isolation and sequencing of the mRNA (host transcriptome) from exfoliated cells has been validated in the context of colon carcinogenesis in rats and humans, and in characterizing human neonatal gastrointestinal development^20–24^. Exfoliated cells, however, have not been used to evaluate a disease affecting the ***small intestine***. Thus, the objective of this study was to determine whether exfoliated cells could be used as a non-invasive method for detecting and studying NSAID enteropathy in a murine model. Specifically, we performed RNA-Seq on colonic and small intestinal mucosa and exfoliated host cells in feces. We then applied computational approaches, e.g., linear discriminant analysis (LDA) and sparse canonical correlation analysis (CCA), to analyze the inter-relatedness of these data. The goals of these studies were to provide proof-of-principle that the exfoliated cell transcriptome (*i*.*e*., the exfoliome) could be used to gain information about NSAID-induced small intestinal injury. Our specific aims were: 1) to determine whether the transcriptome of exfoliated cells reflected gene expression of the small intestinal mucosa; 2) to demonstrate that the transcriptome of exfoliated cells can be used to differentiate healthy and diseased phenotypes; and, 3) to generate hypotheses regarding key biological pathways and processes involved in the pathogenesis of NSAID enteropathy.

## Results

The lumen of the GI tract is a formidable environment for RNA transcripts, particularly the small intestine because of the abundance of both host and microbial enzymes and the longer transit time in the SI relative to the colon. Thus, after extracting RNA from tissues and exfoliated cells we examined RNA quality owing to the potential for degradation of mRNA from exfoliated SI cells in stool as it passes through the GI tract. As expected, Bioanalyzer results revealed lower quality RNA in the exfoliated cells than in the tissue (Figure S1a–c). Bioanalyzer traces show that the majority of RNA in the exfoliated cell samples is of microbial origin (23S and 16S rRNA subunits) (Figure S1b). However, due to the oligo dT probe used in the first step of library construction, the mouse transcripts were selectively targeted for cDNA production and subsequent library content. Fastqc results of the subsequent sequenced transcripts revealed high fidelity and quality with all fecal and tissue samples (Figure S2a,b).

Sequencing of these data revealed that the RNA sequencing reads for SI mucosa, colonic mucosa, and exfoliated cells mapped to an average of 19,324 genes, 20,743 genes, and 13,944 genes per sample, respectively (Figure S3a–c). Genes present in low abundance (*i*.*e*., ≤4 animals or ≤50 times) across all samples were removed from all datasets and the remaining genes subjected to downstream analysis. This reduced the number of transcripts for downstream analysis in the SI to an average of 17,229 genes, the colon data to 17,244 genes, and the exfoliated data to 10,865 genes per sample. Although filtering reduced the total number of genes in the SI and colon (by 11% and 17%, respectively) less than in the exfoliated cells (22%), the total number of reads was negligibly affected in all datasets (SI reduced from 273,065,200 reads across all samples to 273,026,424 – a 0.001% reduction; colon data reduced from 389,222,037 to 389,122,199 - a 0.003% reduction; exfoliated cell data reduced from 38,292,084 to 38,160,589 – a 0.003% reduction) (Figure S3d–f).

**Figure 1:**
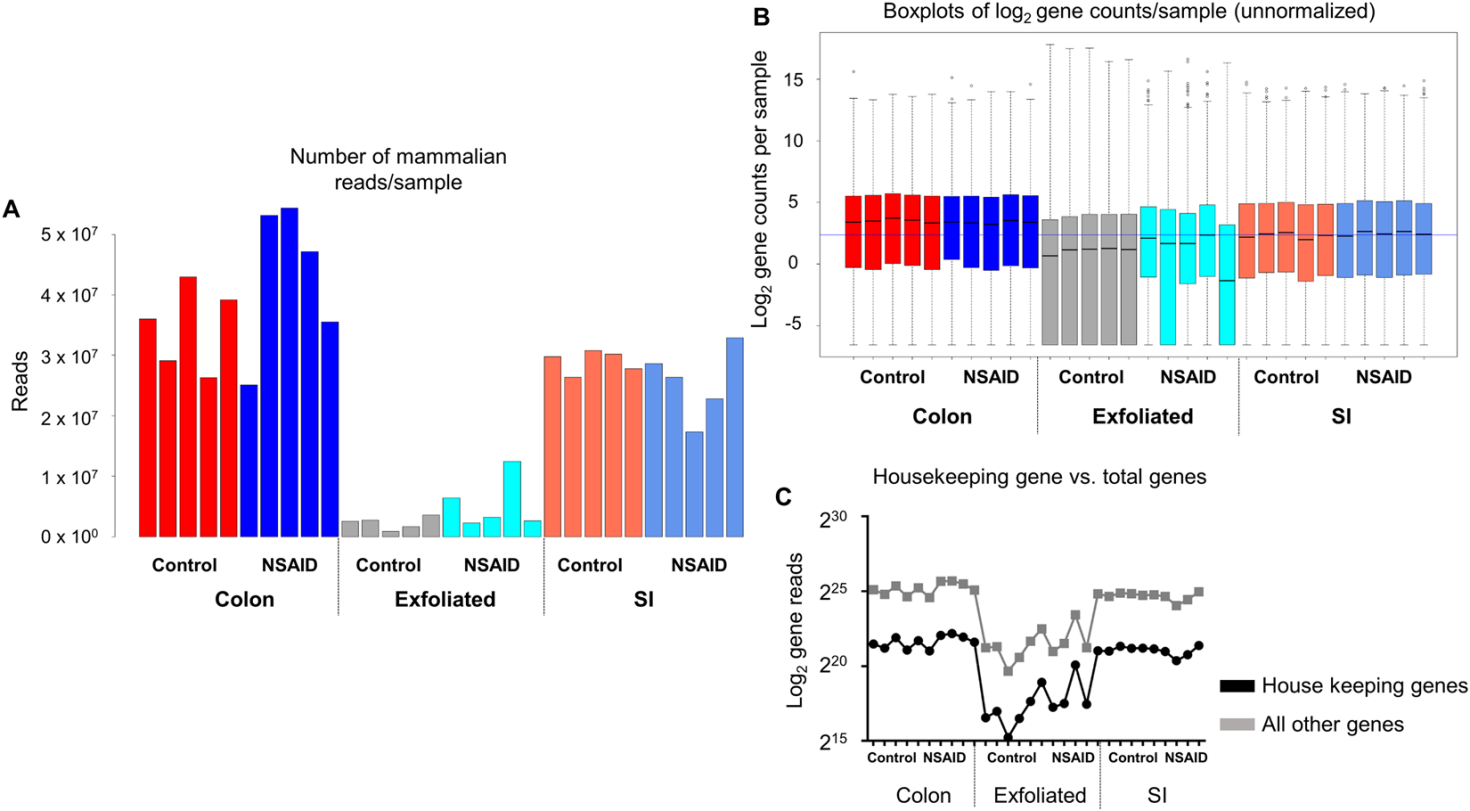
The exfoliome contains fewer reads attributable to the mouse genome than the tissue transcriptomes due to bacterial RNA contamination: (**A**) Number of mammalian reads per sample for each animal and data source colored by treatment group. (**B**) Log(2) counts per gene per sample across all treatment groups from the sequenced RNA colored by treatment group. (**C**) Log(2) total gene reads of 532 murine house-keeping genes (black) and all other genes (grey) per sample across all animals and treatment groups from each data source.

After filtering to remove genes present in low abundance, we examined scatter plots of log(2) counts per million (CPM) for each gene in the 2 treatment groups (*i*.*e*., NSAID and control), comparing the exfoliome to the SI and colonic transcriptome in a pairwise manner (Figure S4a,b). There was strong and significant correlation of the CPM data for each of the pairwise comparisons (Spearman’s correlation coefficient; R value > 0.8 and P < 0.0001 for each pairwise comparison), irrespective of treatment group. We then examined total mammalian gene counts and the number of reads/gene obtained for each sample of the raw, filtered data for all datasets (Fig. 1A,B). There was obvious variation between sources (*i*.*e*., exfoliated cells, SI, or colonic RNA) in total mammalian reads and distribution of counts. This difference was thought to be due to microbial RNA contamination of the RNA extracted from exfoliated cells (Figure S1b) resulting in fewer reads mapping to the mouse genome. To confirm this, we extracted total counts of 532 genes that have been previously identified as housekeeping genes from each sample and plotted those relative to total gene counts^25^. Examination of the relative abundance of these genes in each sample revealed that between-sample total count differences were represented by similar magnitudes of differences in abundance of these 532 house-keeping genes, indicating these differences were due to smaller library size attributable to mammalian transcripts and not to sequencing artifact (Fig. 1C).

**Figure 2:**
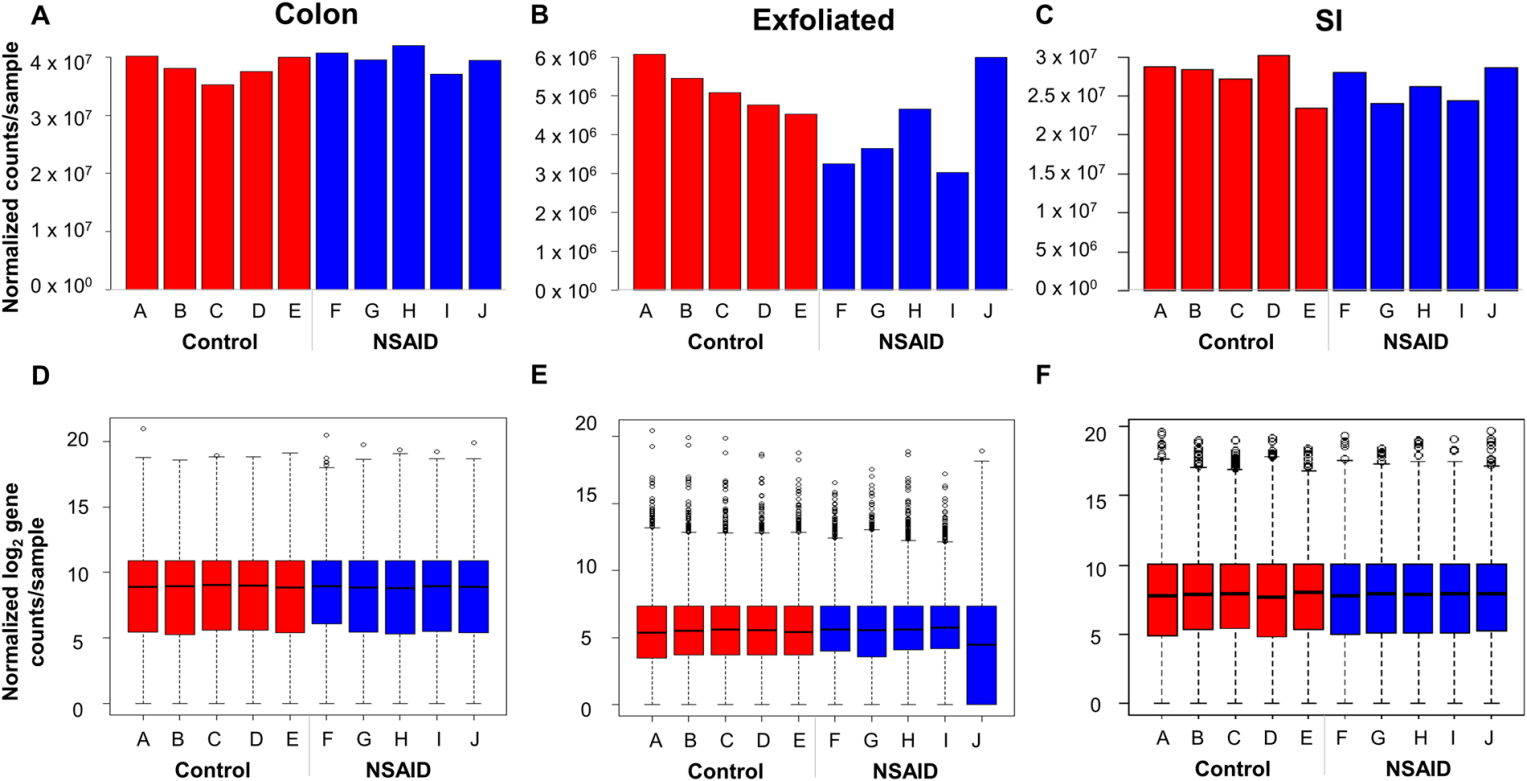
Raw data after filtering and normalization show that the between sample variation in exfoliated cell reads is improved and similar to tissue reads. Total gene counts after normalization for each sample across all treatment groups from the sequenced RNA extracted from (**A**) colonic mucosa, (**B**) exfoliated cells and (**C**) SI mucosa. Normalized log(2) counts per gene per sample across both treatment groups from sequenced RNA extracted from (**D**) colonic mucosa, (**E**) exfoliated cells and (**F**) SI mucosa.

**Figure 3:**
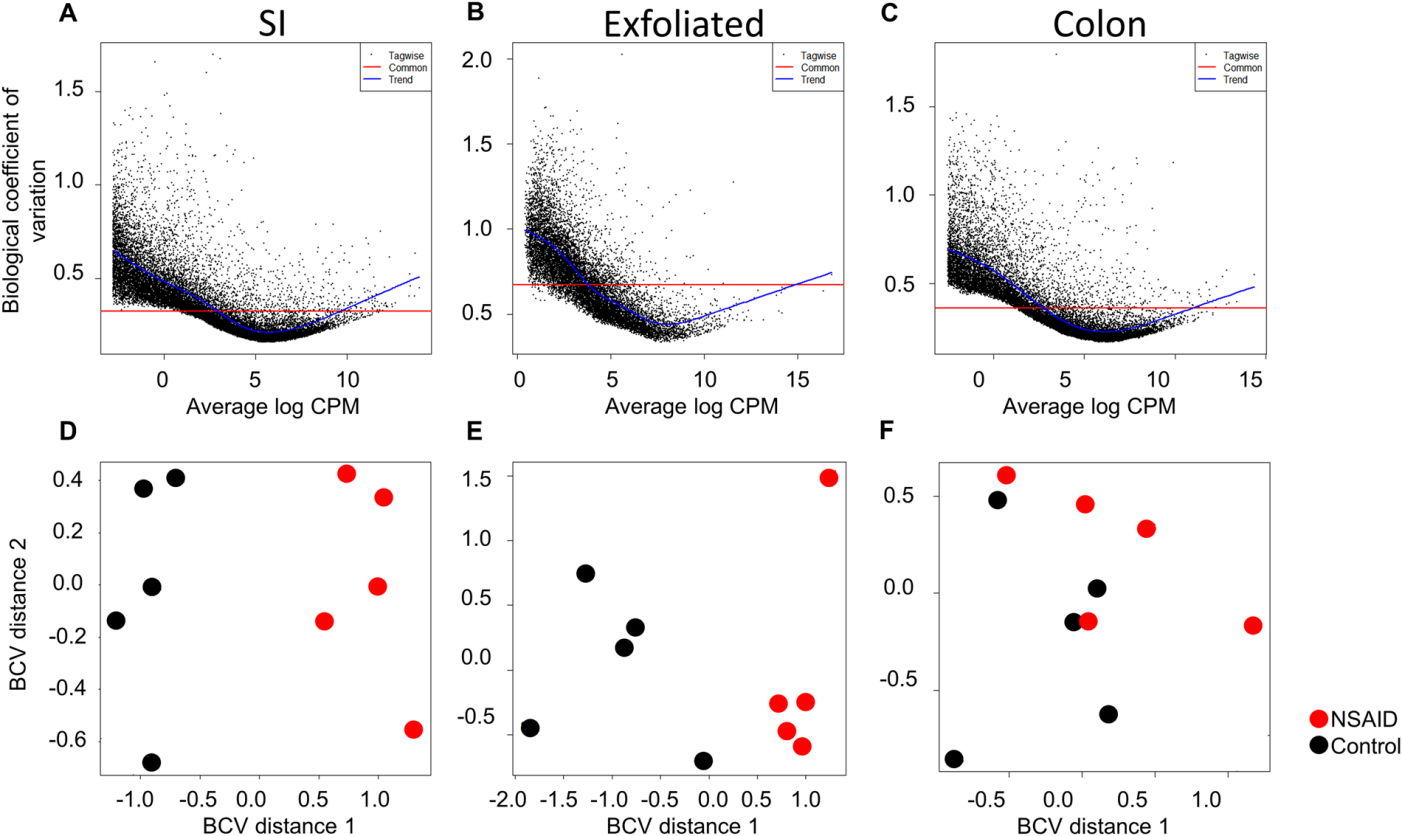
SI transcriptome and exfoliome cluster by treatment group in contrast to colonic transcriptome and biological variability is higher in the exfoliome than the tissue transcriptomes. Biological coefficient of variation (BCV) versus the mean log counts per million (CPM) of the SI transcriptome (**A**), exfoliome (**B**) and colonic transcriptome (**C**). (**D**) Treatment-based multi-dimensional scaling (MDS) plots of the SI transcriptome and that of the exfoliome (**E**) and colon (**F**).

Given the magnitude of differences in library size attributable to mammalian transcripts between the exfoliome and the tissue transciptome, it was necessary to perform the remainder of the analyses separately for each source of RNA (*i*.*e*., SI, colon, or exfoliated cells). To account for between-sample variation in read-counts, RNA-Seq data for each dataset were normalized with edgeR accounting for group effects using the function *calcNormFactors* and the upper-quartile method. Total gene-counts and boxplots of the number of reads/gene for each sample of the normalized data for both tissue and exfoliated cell datasets are shown in Fig. 2A–F. Variability in abundance of post-normalization total house-keeping genes was also improved (Figure S5). Prior to identifying differentially expressed (DE) genes, we assessed biological variability in the exfoliome relative to the tissue transcriptomes. Biological coefficient of variation (BCV) versus the mean log counts per million (CPM) and multi-dimensional scaling (MDS) plots were used to visually assess the similarity of the samples within each treatment group (Fig. 3A–F). These results demonstrate a relatively high degree of variation in the exfoliome (common dispersion = 0.592 and BCV = 0.769) (Fig. 3B) as compared to both tissue transcriptomes (Fig. 3A and C), with a common dispersion of 0.143 and BCV of 0.379 for the SI and common dispersion of 0.126 and BCV of 0.329 for the colon. Notably, MDS based on BCV revealed clear separation of the treatment groups in both the SI transcriptome and exfoliome but not the colonic transcriptome (Fig. 3D–F).

**Figure 4:**
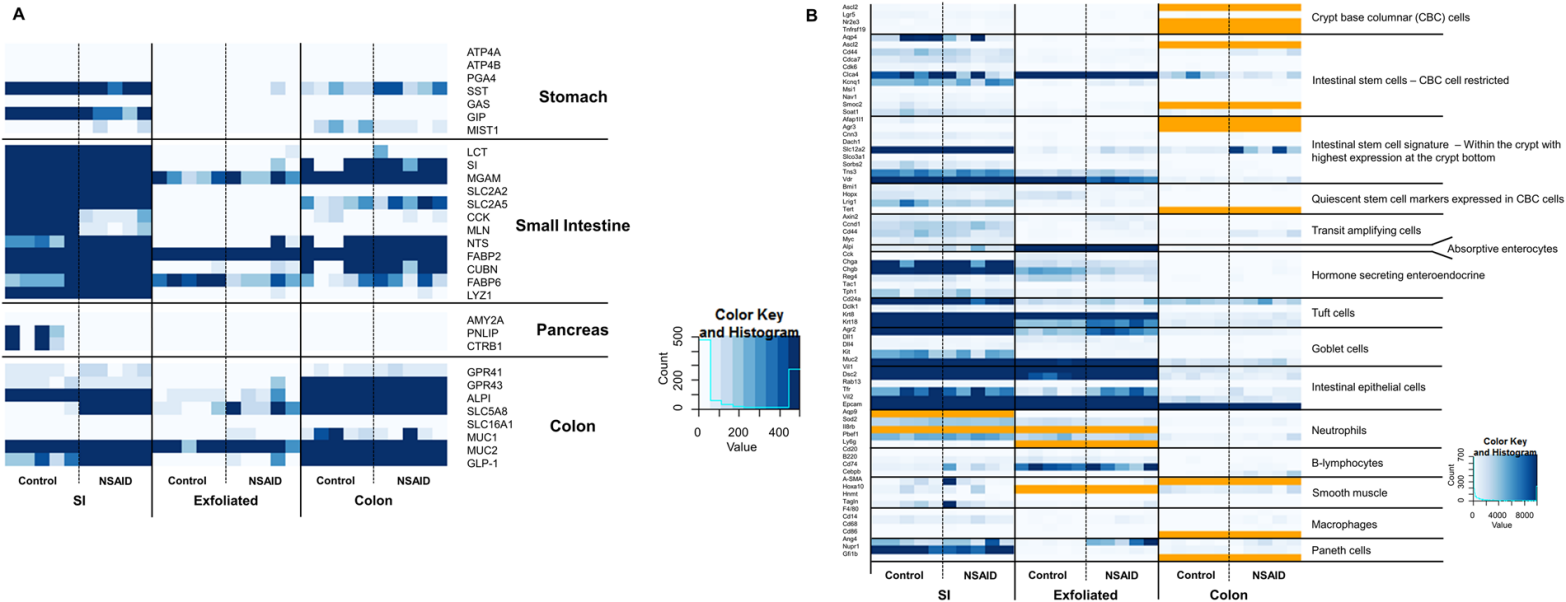
(**A**) The exfoliome signature arises from cells sloughed from both the small intestine and colon and comprises reads from the diverse array of cell types expected to be found in the intestinal mucosa (**A**) Heatmap showing counts of genes that are reported to be primarily expressed at specific anatomic locations (stomach, pancreas, small intestine, colon). All genes with counts greater than 400 are colored dark blue. (**B**) Heatmap showing counts of biomarker genes from each sample and each data source (orange = gene not expressed).

Prior to analyzing these data in order to derive biological meaning, we first wished to determine the anatomic origin of the cellular signature derived from the exfoliome. In order to determine the source of this signature we extracted the counts of genes previously identified and expressed predominantly in specific anatomic locations (*i*.*e*. stomach, pancreas, small intestine, and colon). Interestingly, we found that the exfoliome contained virtually no reads from genes representing the stomach or pancreas. In contrast, there was a clear signature arising from both the colon and small intestine (Fig. 4A). As expected genes representing the colon and small intestine were heavily represented in the transcriptomes arising from those locations with some overlap. Similarly, in addition to anatomic origin we also wished to determine the cell types represented in the exfoliome. Clearly, the intestinal mucosa is comprised not only of IECs but also stem cells, crypt cells, goblet cells, Paneth cells (SI), as well as a host of infiltrating immune cells depending on depth of the sample (*i*.*e*., lamina propria) and disease state of the GI tract (*e*.*g*., inflammation vs. homeostasis). To try to determine the cell types present in these data, we reviewed the literature for marker genes expressed either solely by a specific cell type or at least highly enriched in a specific cell type^26–38^. In particular, we extracted the numbers of reads in each sample across all 3 datasets for the following cell types: intestinal stem cells, IECs, crypt base columnar cells, Paneth cells, tuft cells, goblet cells, macrophages, lymphocytes, neutrophils, and smooth muscle. A list of the genes used as biomarkers for each of these cell types is shown in Table S1. Interestingly, we found that all cell types were present in all datasets as identified by the presence of at least 2 marker genes per cell type (Fig. 4B). Visually there were minimal differences among the 3 datasets with the expected exception of fewer reads in the exfoliated cell data and absence of a few marker genes of intestinal stems cells in the exfoliome with concurrent low expression in the tissue transcriptomes. These data suggest that the mucosal transcriptome and exfoliome represent signatures from similar cell types that comprise not only IECs but reads from the diverse array of cell types expected to be found in the intestinal mucosa.

**Figure 5:**
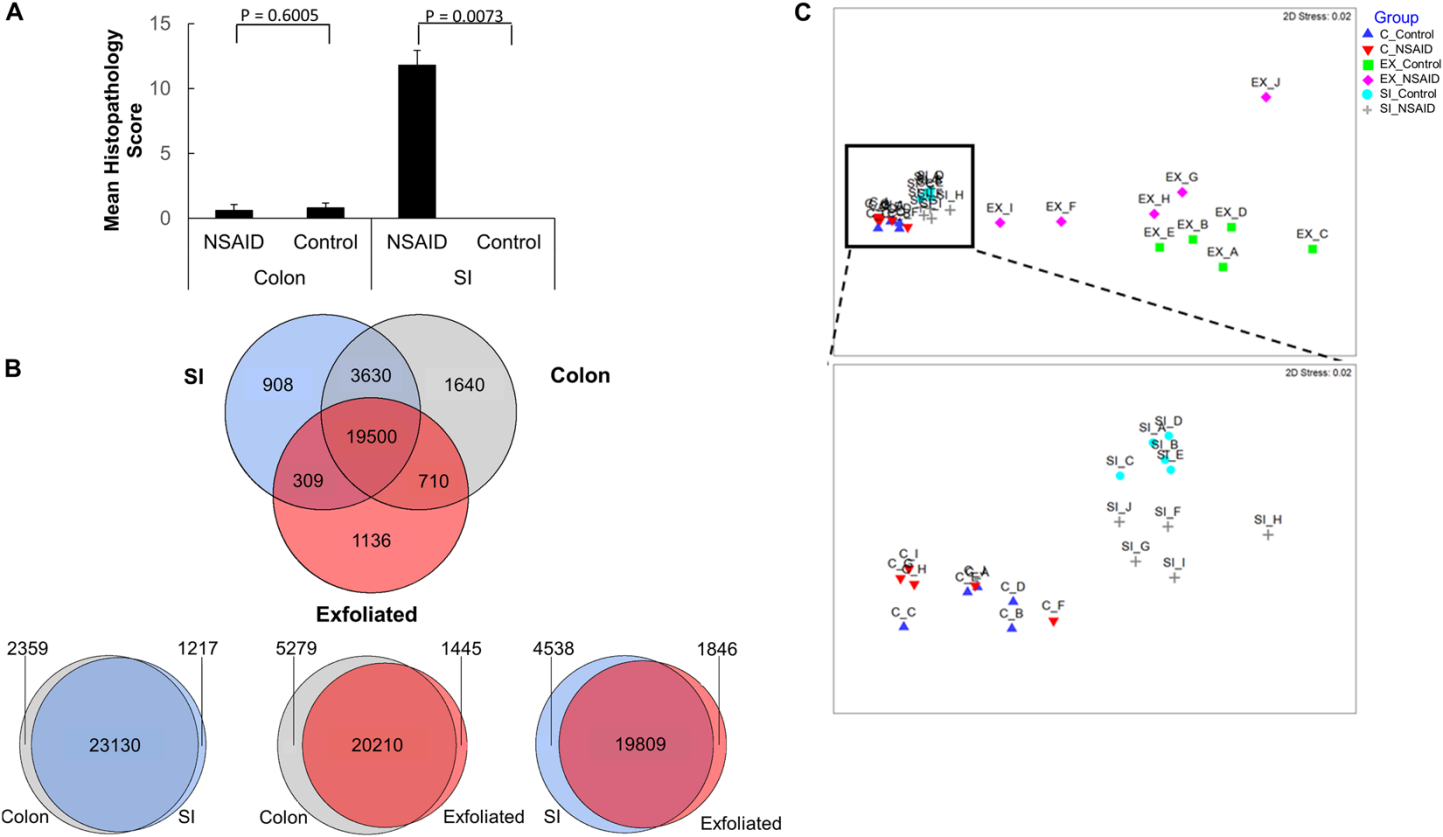
Microscopic pathology reveals NSAID injury is confined to the SI. Despite great overlap between the three RNA-Seq datasets, the exfoliome distinguishes between NSAID and control animals similar to the SI transcriptome, whereas the colonic transcriptome does not. (**A**) Microscopic pathology scores from colon and small intestinal mucosa in control mice and NSAID-treated mice. (**B**) Venn diagram showing intersection of gene lists among the datasets. (**C**) Multi-dimensional scaling plot of each sample color-coded by source and treatment group. Inset of panel C enlarged to show degree of separation of groups in the SI transcriptome and lack of clear separation in colonic transcriptome.

After characterizing the cellular source of the signatures derived from these datasets, we examined each dataset for alterations induced by NSAIDs by comparing the transcriptome (or exfoliome) between the control group and NSAID group. In human subjects and preclinical models, NSAID-induced lower GI damage primarily occurs in the distal jejunum and ileum^3,4^. As expected, we observed marked pathological findings in the distal SI but no notable microscopic abnormalities in the colon in NSAID-treated mice (Fig. 5A). Therefore, RNA extracted from SI mucosal scrapings of NSAID-treated subjects should demonstrate marked mucosal pathology whereas the RNA from colonic mucosal scrapings should reflect minimal pathology.

In order to determine whether the exfoliome more closely resembled the colonic transcriptome or the SI transcriptome, we first crudely examined the intersection of gene lists from each source. This analysis revealed >90% overlap among the 3 datasets in terms of presence and absence of genes (Fig. 5C). Despite this overlap of genes, non-metric multidimensional scaling (NMDS) plots demonstrated differences among these 3 datasets (Fig. 5C and inset). Although the exfoliated cell data differed from the SI and colon data because of smaller library size and fewer mammalian reads potentially resulting from degradation of RNA in the GI tract, evidence of clustering of the treatment groups was observed in the SI and exfoliated cell data but was absent in the colonic data (Fig. 5C and inset). Analysis of similarity (ANOSIM) based on the Bray-Curtis dissimilarity metric quantitatively demonstrated differences between NSAID and control groups for the SI (R value = 0.760; P = 0.008) and exfoliated cells (R value 0.280; P = 0.024) but not for the colonic data (R value 0.194; P = 0.090).

**Figure 6:**
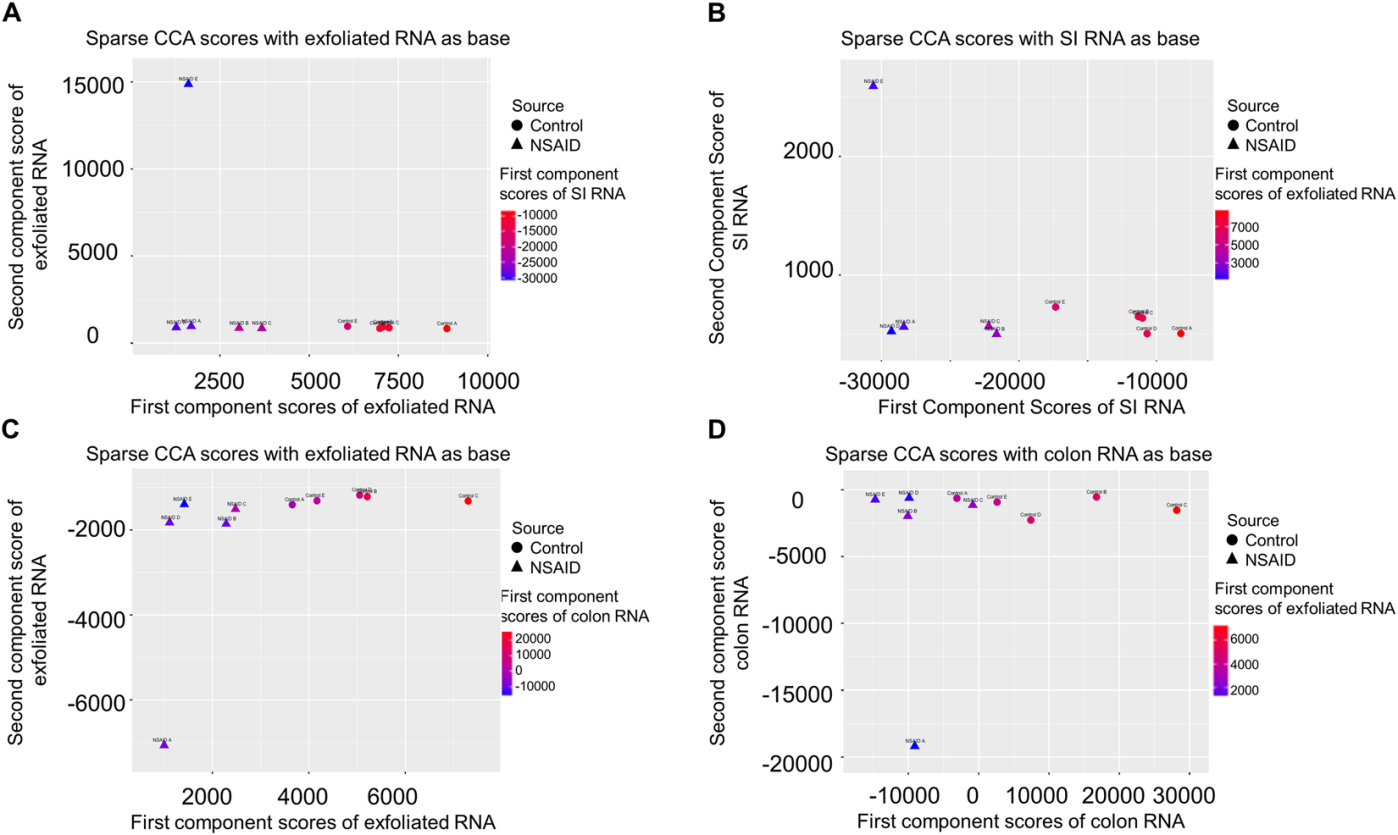
Sparse canonical correlation analysis (CCA) reveals that the global transcriptome profiles from exfoliated cells correlates well with the transcriptome profile of the SI. In contrast, the colonic transcriptome data do not discriminate well between treatment groups. Sparse CCA plots positioned by 1^st^ and 2^nd^ component scores from (exfoliated cells and colored by the 1^st^ component SI scores, (**B**) SI and colored by 1^st^ component scores from exfoliated cells, (**C**) exfoliated cells and colored by the 1^st^ component colon scores and (**D**) colonic mucosa and colored by the 1^st^ component exfoliated scores.

To further examine the interdependent relationship between these 3 transcriptomic profiles, we utilized sparse CCA, a novel multivariate statistical analysis approach. Sparse CCA is a dimensionality reduction technique that identifies the fewest numbers of genes that show the greatest amount of correlation between datasets according to specific optimality criteria. Although the sparse CCA plots should not be assigned any particular biological interpretation, they can be considered a stringent method for determining correlation of large datasets^39^. As revealed by NMDS plots, sparse CCA plots demonstrated that the transcriptome from exfoliated cells correlated well with the SI transcriptome in these mice, and that the SI and exfoliated cell datasets discriminated NSAID-treated from control groups, whereas the colonic mucosal transcriptome did not (Fig. 6A–D).

**Figure 7:**
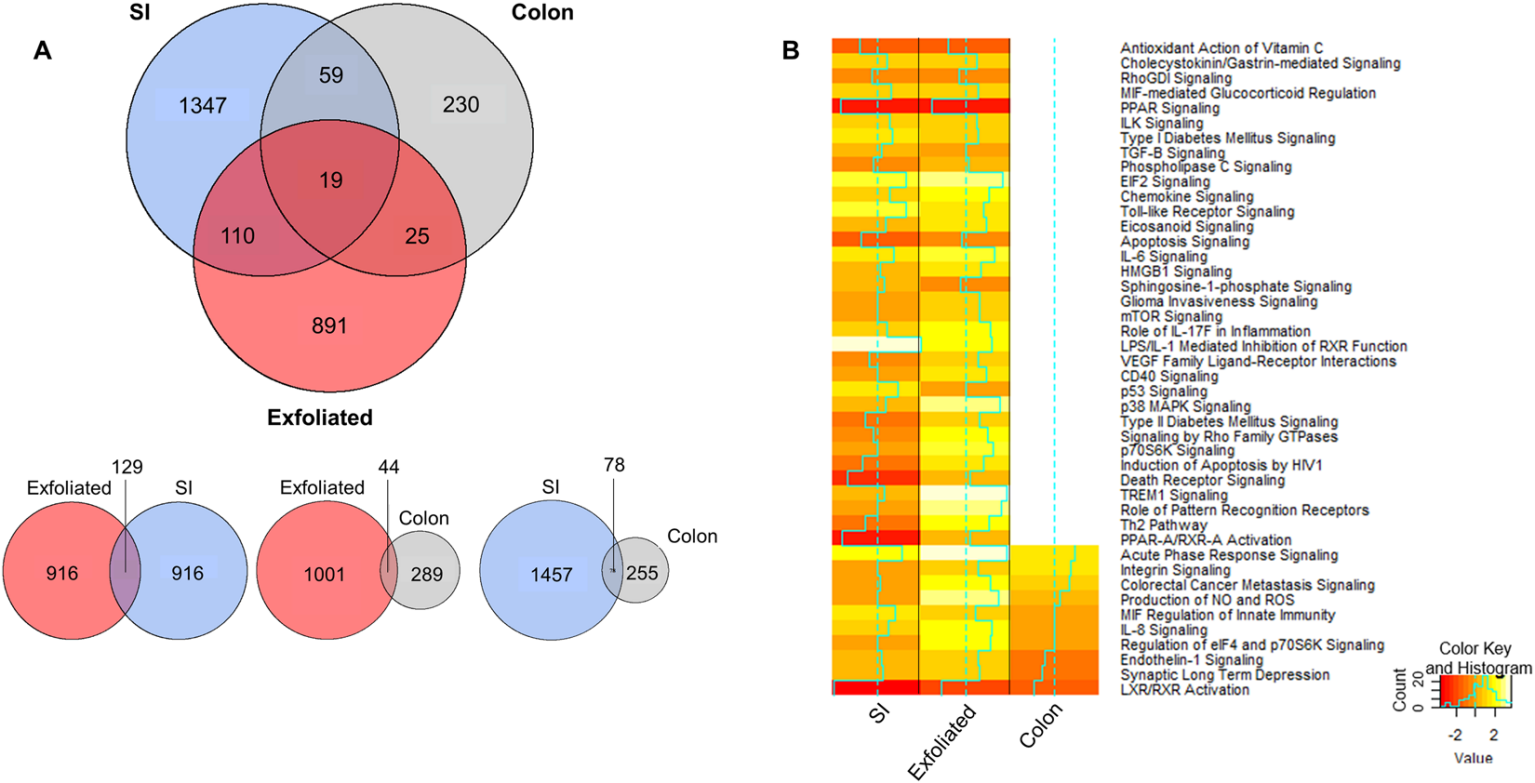
The exfoliated cell transcriptome is similar to the tissue transcriptome as shown by overlapping gene lists and pathways. (**A**) Venn diagram of the intersection of differentially expressed genes found in the exfoliome and tissue transcriptomes. (**B**) Heat map of the Z-scores of the canonical pathways to which the differentially expressed genes between control and NSAID-treated animals were mapped from the SI transcriptome (left column), exfoliome (middle column) and the colonic transcriptome (right column).

**Figure 8:**
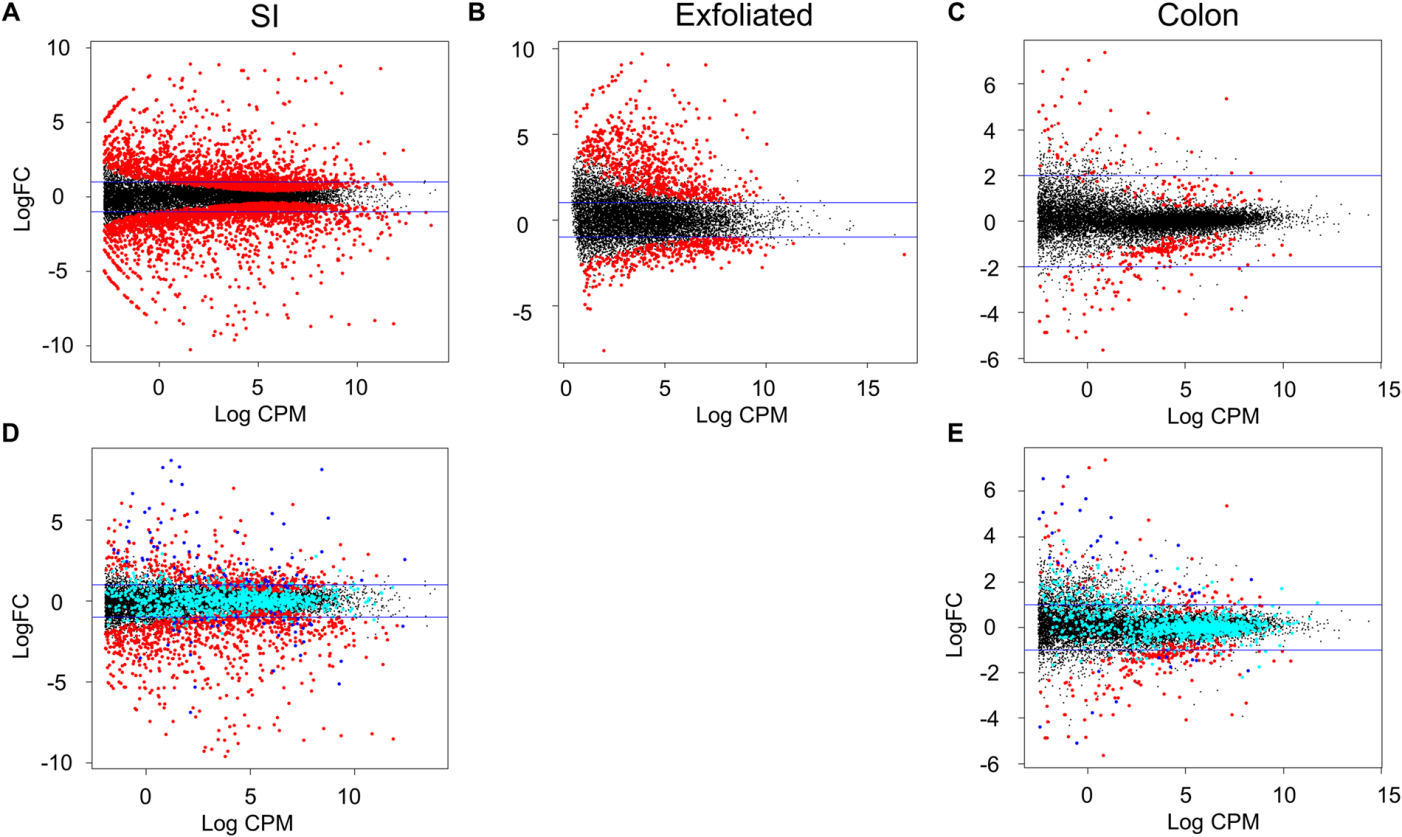
MA plots demonstrate the expression of genes identified as differentially expressed (DE) in the exfoliome and in the tissue transcriptomes. MA plot of genes from the (**A**) SI transcriptome, (**B**) exfoliome and (**C**) colon transcriptome with DE genes (FDR < 0.05) colored red. (**D**) MA plot of genes from the SI transcriptome with genes differentially expressed in the exfoliome AND SI transcriptome colored blue. Genes only differentially expressed in the exfoliome are colored cyan. (**E**) MA plot of genes from the colonic transcriptome with genes differentially expressed in the exfoliome AND colonic transcriptome colored blue. Genes only differentially expressed in the exfoliome are colored cyan.

We next examined the similarities and differences in the gene expression profiles from exfoliated cells compared with those from the scraped intestinal mucosa. We identified DE genes between control and NSAID-treated mice for each dataset. Interestingly, both the exfoliome and SI transcriptome had >1000 DE genes (FDR P < 0.05) between control and NSAID-treated mice, whereas there were relatively few DE genes (n = 333 genes) in the colonic transcriptome (Fig. 7A). Venn diagrams revealed sparse overlap (12%) of the DE expressed genes between the SI transcriptome and the exfoliome and even less overlap between the colonic transcriptome and the exfoliome (6%; Fig. 7A). Despite this sparse overlap, the pathways enriched in the exfoliome and SI transcriptome were similar whereas there was much less similarity between the exfoliome and colonic transcriptome. Specifically, IPA Ingenuity Knowledgebase (www.ingenuity.com) pathway analysis revealed both SI and exfoliated cell datasets exhibited similar occupancy and predicted directionality (Z-score) in the canonical pathways represented (Fig. 7B). In contrast, few pathways were represented in the colonic data (Fig. 7B). For example, Toll-like receptor signaling (which is known to play a crucial role in the pathogenesis of NSAID enteropathy)^9,10,13,18,40,41^ was upregulated by NSAID administration in both the exfoliome and small intestinal transcriptome but genes related to this pathway were not altered in the colonic transcriptome resulting in no occupancy of this canonical pathway. Indeed, the proportion of pathways represented in the colon (31%; 21/67) was significantly less than that of the exfoliome (84%; 56/67; P < 0.0001) and the SI (93%; 62/67; P < 0.0001). Moreover, Pearson’s correlation revealed significant correlation of the Z scores from the exfoliome canonical pathways with those of the SI transcriptome (R = 0.3468; P = 0.0110). Further, McNemar’s test revealed significant (P < 0.0001) discordance in pathways represented between colon and SI and colon and exfoliated cells, but not between SI and exfoliated cells (P = 0.149). A similar finding was observed when examining upstream regulators of these pathways (Figure S6). Specifically, there were significantly fewer upstream regulators expressed in the colon (41%; 780/1888) than in either the SI (73%; 1380/1888; P < 0.0001) or exfoliome (78%; 1469/1888; P < 0.0001). When comparing the upstream regulators expressed in both SI and exfoliated cells, there was significant correlation between the fold-changes in SI and exfoliome gene expression (R = 0.616; P < 0.001). In order to determine the correlation between expression levels in exfoliated cells and the tissues and to demonstrate effect sizes of the differences in gene expression, MA plots for each dataset were constructed and colored to show where the DE genes in the exfoliome resided in both tissues (Fig. 8A–E)^42^. These plots confirmed lack of overlap between DE genes within each dataset and show that the DE genes within the exfoliome had a greater effect-size than those within the tissue.

**Figure 9:**
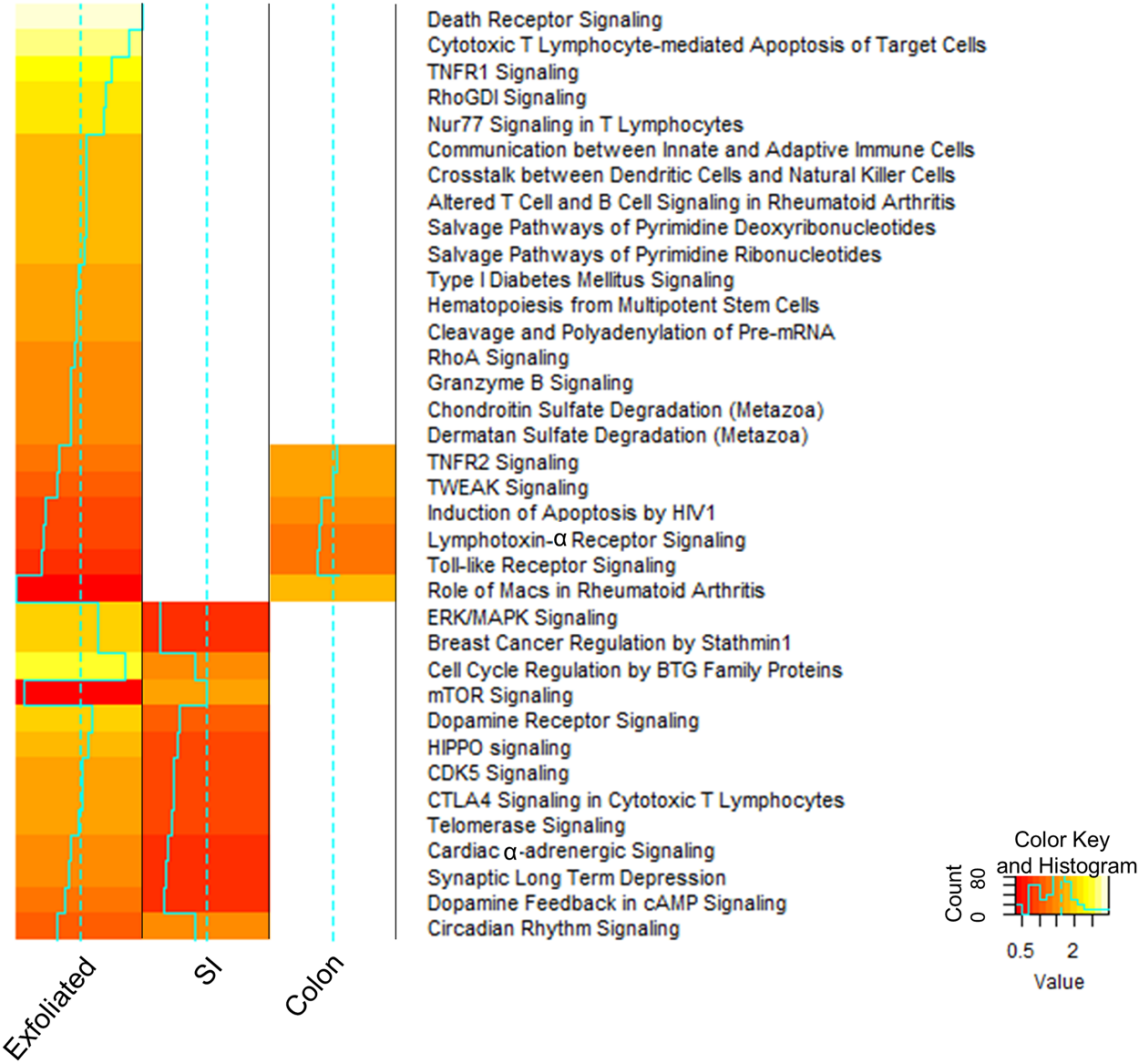
1-Feature linear discriminate analysis (LDA) substantiates the ability of both the SI transcriptome and exfoliome to differentiate between control and NSAID-treated animals. Heatmap showing Z-scores of canonical pathways identified by 1-feature LDA of genes identified as being DE (FDR < 0.05) in each dataset.

The ability of both the SI transcriptome and exfoliome to differentiate between control and NSAID-treated animals was further substantiated by LDA of the list of differentially expressed genes between treatment groups in the 3 datasets to identify the most biologically-relevant genes. LDA was performed on the counts of DE genes identified from the exfoliome, SI transcriptome, and colonic transcriptome. The 1-feature (*i*.*e*., single gene) LDA identified 34 and 49 genes from the SI and exfoliated datasets, respectively, that differentiated control from NSAID-treated animals with less than 5% error, but only 17 genes from the colonic data (Table S2). These genes were subsequently uploaded into IPA along with their respective P-values and fold-changes in order to determine which pathways identified by LDA could best classify NSAID-treated versus control mice with respect to all differentially expressed genes. The top (lowest P-value) upstream regulators and the networks to which these genes belong are shown in Fig. 9.

## Discussion

Here we have shown that the transcriptome of exfoliated cells closely resembles that at the affected tissue level in a murine model of NSAID enteropathy. Using RNA-Seq, we compared the exfoliome to the transcriptome of mucosa from the SI and colon. Although gene expression of exfoliated cells has been used to examine GI development in neonates and for biomarkers of colorectal cancer, we show for the first time the application of this approach to examine a disease that primarily affects the SI^2,9,13,14,43^. The use of exfoliated cell mRNA presents several important advantages relative to mRNA obtained from the standard approach of tissue biopsy. First, use of fecal samples is non-invasive and results in no physical pain to the patient. Second, greater requirement of time, effort, and cost is required for collection of biopsy specimens than for collection of feces. Third, although the primary area of injury with NSAID enteropathy is the distal small intestine, NSAIDs can affect both the large and small intestine of humans and other animals^44,45^. Exfoliated cells arise from both the colon and SI and thus provide an overview of the gene expression profiles of the entire GI tract, whereas a tissue specimen only provides a site-specific gene expression profile^21^. Fourth, intestinal biopsies generally can be collected once (typically at a terminal stage of the study) in mice, whereas feces can be collected sequentially, thereby allowing the temporality of gene expression profiles from exfoliated cells to be used to investigate and monitor disease progression. If the same findings hold true in humans, fecal samples could be used as a non-invasive tool for detecting and monitoring patients with NSAID enteropathy.

Concerns regarding the use of mammalian RNA found in voided stool are valid given the likelihood of RNA degradation as it passes through the GI tract. Indeed, we observed differences in the quality of RNA found in stool versus mucosa. It appears that appropriate selection of polyA tails and utilization of purification steps outlined in this manuscript results in RNA that is suitable for sequencing. Based on both the results of the quality control measures utilized here and the similarity of the signature in the exfoliome versus the tissue transcriptomes, results obtained by sequencing the exfoliome can be accurate and informative. An additional aspect that might have improved quality scores is the fact that we performed 50-bp, single-end sequencing because longer transcripts likely underwent further degradation as they passed through the GI tract, which could have resulted in lower quality scores for the bases located beyond the 50-bp segments sequenced in our study.

In general, the exfoliome correlated well with the tissue transcriptome as evidenced by the intersection of genes present in all datasets. From a clinical perspective, the exfoliome was similar to the SI mucosal transcriptome, and discriminated NSAID-treated from control mice similarly to mucosa from the ileum, the region of the GI tract most severely affected in NSAID enteropathy in both mice and human subjects^40,46–49^. In contrast, the transcriptome of the colonic mucosa, which sustained less injury due to NSAID treatment, failed to discriminate between treatment groups. These results are interesting because much of the RNA extracted from exfoliome undoubtedly originates from the colon. Although we did not attempt to identify the precise source of these cells, marker genes representing various anatomic locations within the GI tract (*i*.*e*., stomach, small intestine, pancreas, and colon) revealed that the signature in the exfoliome was derived from both the small intestine and the colon with virtually no signature coming from the stomach or pancreas. Additionally, the many cell types that reside in the mucosa and lamina propria were present in the exfoliome (and tissue), indicating that these data represent many cell types. It is impossible to determine the exact source of each transcript found in the exfoliome and this may explain, in part, the lack of overlap between DE genes between the exfoliome and SI transcriptomes. For example, inflammatory cytokines are expressed following NSAID- induced impairment of barrier function and resultant influx of LPS and other components of the microbiome^16,50,51^. The many cell types found in the intestinal mucosa are then exposed to these cytokines and each cell type may behave differently following exposure to these cytokines. One example of such differing behavior in response to similar stimulation is the dichotomy of the effects of macrophages and neutrophils in response to intestinal inflammation whereby macrophages dampen the effects of neutrophilic inflammation in the gut^52^. This could explain similar pathway analysis results arising from lists of DE genes that do not have substantial overlap. Nonetheless, the ability of the exfoliome to discriminate between control and NSAID-treated mice in a manner similar to the SI transcriptome indicates that studying the exfoliome can yield informative results about the distal small intestine irrespective of knowledge of the precise source of the exfoliated cells. Further investigation of the impact of concomitant colonic disease and other potential confounding factors on use of the exfoliated cell transcriptome is merited.

Having demonstrated that the exfoliome reflected the SI transcriptome, we used gene expression data to examine which pathways and processes were implicated in NSAID enteropathy. Our results further substantiate the importance of innate immune responses in NSAID enteropathy^53,54^. Specifically, and as reported previously, we identified several pro-inflammatory pathways and upstream regulators including IL-8 (mouse homologs), IL-17, NF-KB, IL-1, and MYD88 as being involved in NSAID enteropathy^13,40,53–55^. Toll-like receptor (TLR) signaling and specifically TLR-4 is known to be a critical component of the pathophysiology of NSAID enteropathy^40,41^. As expected, this pathway was highly up-regulated in both the SI transcriptome and the exfoliome but expression of the genes belonging to this pathway were not altered in the colonic mucosa and therefore this pathway showed no occupancy in the data arising from the colonic mucosa. The lack of occupancy of this and other pathways crucial to the pathogenesis of NSAID enteropathy in the colonic data, including apoptosis, and IL-1 and IL-6 pathways, provide further evidence that the exfoliome signature reflects the SI mucosa in the presence of SI disease. The consistency of our exfoliome results with those of prior studies implicating innate immunity as a critical player in NSAID enteropathy adds evidence to the validity of using this novel approach for the study of NSAID enteropathy (and potentially numerous other small intestinal disorders).

There were several limitations to this study. First, the evaluated sample size was small and consisted of a single time-point (after 7 days of NSAID treatment). Despite the small sample size, we clearly demonstrated that the exfoliated cell transcriptome reflects that of the tissue at the level of the ileum. In addition, our approach revealed the importance of several pathways involved in both NSAID enteropathy. Therefore, even with a small sample size, we were able to recapitulate known biologically relevant findings with this technique. In the latter or more severe stages of NSAID enteropathy, the major pathways involved are the host response to increased mucosal permeability. Therefore, to better understand the temporal events contributing to NSAID enteropathy, a more appropriate approach might be to examine earlier time points.

In summary, we have demonstrated that the exfoliated cell transcriptome closely mirrors the transcriptome of the tissue at the level of the ileum. Although use of the exfoliome has been described previously, this is the first time these findings have been correlated to the tissue-level transcriptome. In addition, this technique has not been utilized to examine a disease that primarily affects the SI. We have shown that the exfoliated cell transcriptome can be a useful tool to examine NSAID enteropathy and potentially extend to a number of other GI diseases, including those affecting the SI. Additionally, the application of noninvasive exfoliated cell-based techniques might be useful for the early identification and monitoring of therapeutic interventions targeting NSAID enteropathy and other intestinal disorders.

## Methods

### Animals and sample collection

Animal protocols were approved by the Texas A&M Institutional Animal Care and Use Committee in accordance with appropriate institutional and regulatory bodies’ guidelines. Animals were handled and treated as previously described^43^. Briefly, 8- to 10-week-old, specific-pathogen-free female C57BL/6 J mice were purchased and allowed to acclimate for 2 weeks. Mice were fed standardized laboratory rodent diet (Teklad rodent diet, Envigo product number 8604) and sterile water *ad libitum*. Mice were randomly divided into 2 groups (n = 5 mice/group): NSAID (indomethacin) or control (vehicle). Mice were then rehoused on the basis of treatment group assignment, with 5 animals/group-cage. To induce NSAID enteropathy, mice in the NSAID group were gavaged once daily with indomethacin (5 mg/kg; for 7 days; Sigma Aldrich, St. Louis, MO) dissolved in DMSO (Sigma Aldrich, St. Louis, MO) and further diluted in phosphate buffered saline (PBS). Mice in the control group were gavaged with equal volumes (200 μL) of PBS including an equal concentration of DMSO (0.001%). Treatments occurred between 9 AM and 11 AM CST once daily for 7 days. Feces were collected daily by placing individual animals in sterile plastic cups that were RNase- and DNase-free until they passed feces. The mice were returned to their home cages and the feces immediately placed in 500 µL denaturation solution (ThermoFisher Scientific), homogenized and flash frozen at −80 °C. On day 7, the mice were euthanized via CO_2_ asphyxiation. The small intestine and colon were removed, flushed with ice-cold PBS, fixed in 4% paraformaldehyde, Swiss-rolled, paraffin-embedded, and stained with hematoxylin and eosin. Stained sections of the small intestine were scored for intestinal inflammation as previously described^56^.

### Extraction of mRNA from exfoliated cells and library preparation

PolyA^+^ RNA was isolated from stool samples from mice as previously described^24,57^. RNA was quantified using a Nanodrop spectrophotometer (Thermo Fisher Scientific, Waltham, MA) and the quality was assessed using the Nano6000 chip on a Bioanalyzer 2100 (Agilent Technologies, Santa Clara, CA). Each mouse sample was processed with the NuGEN Ovation 3′-DGE kit (San Carlos, CA) to convert RNA into cDNA followed by NuGEN Encore Rapid Library kit to create Illumina libraries, as per manufacturer’s instructions. Briefly, 30 ng of each sample were used to synthesize first and second strand cDNA, followed by purification using Agencourt RNAClean XP beads (Brea, CA) included in the kit. The cDNA was linearly amplified using the NuGEN SPIA primer, and cDNA quantity was determined using Nanodrop spectrophotometer (Thermo Fisher Scientific). Two micrograms of cDNA were fragmented using a Covaris S2 sonicator (Woburn, MA) with the following settings: duty cycle 10%, intensity 5, cycles/burst 100, and time 5 min. Fragmented samples were concentrated using the MinElute Reaction Cleanup kit (Qiagen, Germantown, MD) as per manufacturer’s instructions. Samples were subsequently quantified using a Nanodrop spectrophotometer. Following cDNA fragment repair and purification, Illumina adaptors were ligated onto fragment ends and amplified to create the final library. Libraries were quantified using the Kapa Library Quantification kit (Kapa Biosystems-Sigma Aldrich, St. Louis, MO) and run on an Agilent DNA High Sensitivity Chip to confirm appropriate sizing and the exclusion of adapter dimers.

### Tissue mRNA extraction and library preparation

RNA was extracted from ileal and colonic mucosal scrapings using an RNeasy mini kit (QIAGEN, Redwood City, CA) following the manufacturer’s instructions and including on-column DNase treatment. RNA quantity was determined using a Nanodrop spectrophotometer (Thermo Fisher Scientific) and the quality was assessed using the Nano6000 chip on a Bioanalyzer 2100 (Agilent Technologies). The samples were randomized prior to RNA-Seq library preparation. Sequencing libraries were made using 250 ng of RNA and the TruSeq RNA Sample Preparation kit (Illumina) following the manufacturer’s instructions.

### RNA sequencing and downstream processing

Libraries were pooled and sequenced on an Illumina HiSeq. 2500 at the Texas AgriLife Genomics and Bioinformatics Services Core Facility (College Station, TX). Sequence data were uploaded into NCBI small reads archive (Accession number PRJNA290483). Sequencing data were provided in a de-multiplexed format and aligned using Spliced Transcripts Alignment to a Reference (STAR) software with default parameters and referenced against the genome of *Mus musculus* (Ensembl version *GRCm38*)^58^. Differentially expressed genes were determined using EdgeR based on the matrix of gene counts^42^. The open source package edgeR was selected based on two considerations: first, it uses the state-of-the art general linear model framework for detection of differentially expressed genes and second, there is evidence that it performs well relative to other tools for differential gene expression^59–61^. Gene pathway involvement and intersections were analyzed using QIAGEN’s Ingenuity® Pathway Analysis (IPA, QIAGEN, Redwood City, CA) by uploading appropriate gene lists with fold-change and false discovery rate (FDR) P-values.

### Sparse canonical correlation analysis (CCA)

In order to take into account the multivariate structure when assessing and ranking genes, the multivariate relationships between the exfoliated cell and tissue transcriptomic datasets were analytically quantified. Sparse CCA provides measures of the strength of multivariate association between variable sets as well as a means to interpret the role of variables in terms of the underlying multivariate relationship^23^. Briefly, for the 2 random vectors ${\rm{x}}={({x}_{1},{x}_{2},\ldots ,{x}_{p})}^{T}$ and ${\rm{y}}={({y}_{1},{y}_{2},\ldots ,{y}_{q})}^{T}$ CCA aims to find 2 projection directions ${u}_{1}\in {{\mathbb{R}}}^{p}$ and ${v}_{1}\in {{\mathbb{R}}}^{q}$ so that;

1 $$({u}_{1},{v}_{1})=\mathop{{\rm{argmax}}}\limits_{u,v}\,{\rm{Corr}}({u}^{T}x,{v}^{T}y)=\mathop{{\rm{argmax}}}\limits_{u,v}\frac{{u}^{T}{{\rm{\Sigma }}}_{xy}\nu }{\sqrt{({u}^{T}{{\rm{\Sigma }}}_{xx}\nu )({v}^{T}{{\rm{\Sigma }}}_{yy}\nu )}},$$

where, ${{\rm{\Sigma }}}_{xy},{{\rm{\Sigma }}}_{xx},{{\rm{\Sigma }}}_{yy}$ are covariance and variance matrices. Let $X$ and $Y$ denote $n\times p\,\,$and $n\times q$ data matrices as the observations from random vectors $x$ and $y$, respectively. Thus, the empirical version of the above objective function can be written as;

2 $$\begin{array}{cc}\mathop{{\rm{\max }}}\limits_{u,v} & {u}^{T}{X}^{T}Yv\\ {\rm{subject}}\,{\rm{to}} & {u}^{T}\frac{{X}^{T}X}{n}u=1,\,{v}^{T}\frac{{Y}^{T}Y}{n}v=\,1.\end{array}$$

This objective function, however, does not have a closed-form solution when the sample size $n$ is less than ${\rm{\min }}(p,q)$. In addition, the result does not perform variable selection and hence there could be problems with the biological interpretability. To overcome these limitations, sparse CCA adds regularization conditions and obtains a sparse solution with a reduced computational cost^39,62,63^. The main idea of this approach is to utilize;

3 $$\begin{array}{cc}\mathop{{\rm{\max }}}\limits_{u,v} & {u}^{T}{X}^{T}Yv\\ {\rm{subject}}\,{\rm{to}} & \begin{array}{c}{u}^{T}{X}^{T}Xu\le 1,\,{v}^{T}{Y}^{T}Yv\le 1\\ \parallel u{\parallel }_{1}\le {c}_{1},\,\parallel v{\parallel }_{1}\le {c}_{2},\end{array}\end{array}$$

where the tuning parameters ${c}_{1}$, ${c}_{2}$, are positive. It has been shown that in high-dimensional problems, treating the covariance matrix as a diagonal one yields satisfactory results^39,62^. Thus;

4 $$\begin{array}{cc}\mathop{{\rm{\max }}}\limits_{u,v} & {u}^{T}{X}^{T}Yv\\ {\rm{subject}}\,{\rm{to}} & \begin{array}{c}\parallel v{\parallel }_{2}\le 1,\,\parallel u{\parallel }_{2}\le 1,\\ \parallel u{\parallel }_{1}\le {c}_{1},\,\parallel v{\parallel }_{1}\le {c}_{2},\end{array}\end{array}$$

yields the first pair of sparse canonical correlation loadings. The algorithm to solve this optimization problem uses soft-thresholding, binary search, and finds the second pair of sparse canonical correlation components via the deflation method^62,64^. In our implementation of the sparse CCA method, a pair of tuning parameters $({c}_{1},{c}_{2})$ with values between $0$ and $1$ were utilized. To select the tuning parameters, the leave-one-out cross-validation was used. Specifically, a grid of dense values from $(0,1)\times (0,1)$ were used as potential values of $({c}_{1},{c}_{2})$ and the absolute value of correlation without penalty was calculated as the criterion function in a cross validation method.

### Linear discriminant analysis (LDA)

We used the edgeR output of genes that were found to be differentially expressed between NSAID and control animals with a false discovery rate (FDR) P value of < 0.05 for feature set identification. Subsequently, we used a previously described method to determine, within the differentially expressed genes, which genes (1-feature) accurately discriminated NSAID-treated animals from control animals as given by the classification error rates^21^. Estimation of the classification error is of critical importance when the number of potential feature sets is large. When sample size is limited, an error estimator may have a large variance and, therefore, may often be low, even if it is approximately unbiased. This can produce many feature sets and classifiers with low error estimates. In this case, the problem was mitigated by applying a bolstered error estimation technique^21,65^. The result of this approach is a list of feature sets that are ranked with respect to their bolstered classification error estimates.

### Availability of Data and Materials

The datasets utilized for the current study are available via the NCBI bioproject (Accession number PRJNA290483) http://www.ncbi.nlm.nih.gov/bioproject/.

## Electronic supplementary material

Supplementary Information

## References

[1] Wolfe MM, Lichtenstein DR, Singh G (1999). Gastrointestinal toxicity of nonsteroidal antiinflammatory drugs. N Engl J Med.

[2] Graham DY, Opekun AR, Willingham FF, Qureshi WA (2005). Visible small-intestinal mucosal injury in chronic NSAID users. Clinical gastroenterology and hepatology: the official clinical practice journal of the American Gastroenterological Association.

[3] Becker JC, Domschke W, Pohle T (2004). Current approaches to prevent NSAID-induced gastropathy–COX selectivity and beyond. British journal of clinical pharmacology.

[4] Wallace JL (2013). Mechanisms, prevention and clinical implications of nonsteroidal anti-inflammatory drug-enteropathy. World journal of gastroenterology.

[5] Shaheen NJ, Straus WL, Sandler RS (2002). Chemoprevention of gastrointestinal malignancies with nonsteroidal antiinflammatory drugs. Cancer.

[6] Cohen ND, Carter GK, Mealey RH, Taylor TS (1995). Medical management of right dorsal colitis in 5 horses: a retrospective study (1987–1993). Journal of veterinary internal medicine.

[7] McConnico RS, Morgan TW, Williams CC, Hubert JD, Moore RM (2008). Pathophysiologic effects of phenylbutazone on the right dorsal colon in horses. American journal of veterinary research.

[8] Marshall, J. F. & Blikslager, A. T. The effect of nonsteroidal anti-inflammatory drugs on the equine intestine. *Equine veterinary journal*. *Supplement*, 140–144, 10.1111/j.2042-3306.2011.00398.x (2011). 10.1111/j.2042-3306.2011.00398.x 21790769

[9] Wallace JL (2012). NSAID gastropathy and enteropathy: distinct pathogenesis likely necessitates distinct prevention strategies. Br J Pharmacol.

[10] Handa O, Naito Y, Fukui A, Omatsu T, Yoshikawa T (2014). The impact of non-steroidal anti-inflammatory drugs on the small intestinal epithelium. Journal of clinical biochemistry and nutrition.

[11] Santucci L (1994). Pentoxifylline prevents indomethacin induced acute gastric mucosal damage in rats: role of tumour necrosis factor alpha. Gut.

[12] Appleyard CB, McCafferty DM, Tigley AW, Swain MG, Wallace JL (1996). Tumor necrosis factor mediation of NSAID-induced gastric damage: role of leukocyte adherence. The American journal of physiology.

[13] Watanabe T (2008). Non-steroidal anti-inflammatory drug-induced small intestinal damage is Toll-like receptor 4 dependent. Gut.

[14] Wallace, J. L. *et al*. Proton pump inhibitors exacerbate NSAID-induced small intestinal injury by inducing dysbiosis. *Gastroenterology* **141**, 1314–1322, 1322. e1311–1315, 10.1053/j.gastro.2011.06.075 (2011). 10.1053/j.gastro.2011.06.075 21745447

[15] Makivuokko H, Tiihonen K, Tynkkynen S, Paulin L, Rautonen N (2010). The effect of age and non-steroidal anti-inflammatory drugs on human intestinal microbiota composition. The British journal of nutrition.

[16] Hagiwara M, Kataoka K, Arimochi H, Kuwahara T, Ohnishi Y (2004). Role of unbalanced growth of gram-negative bacteria in ileal ulcer formation in rats treated with a nonsteroidal anti-inflammatory drug. The journal of medical investigation: JMI.

[17] Uejima M, Kinouchi T, Kataoka K, Hiraoka I, Ohnishi Y (1996). Role of intestinal bacteria in ileal ulcer formation in rats treated with a nonsteroidal antiinflammatory drug. Microbiology and immunology.

[18] Koga H, Aoyagi K, Matsumoto T, Iida M, Fujishima M (1999). Experimental enteropathy in athymic and euthymic rats: synergistic role of lipopolysaccharide and indomethacin. The American journal of physiology.

[19] Potten CS, Schofield R, Lajtha LG (1979). A comparison of cell replacement in bone marrow, testis and three regions of surface epithelium. Biochimica et biophysica acta.

[20] Chapkin RS (1998). Dietary fiber differentially alters cellular fatty acid-binding protein expression in exfoliated colonocytes during tumor development. Nutrition and cancer.

[21] Chapkin RS (2010). Noninvasive stool-based detection of infant gastrointestinal development using gene expression profiles from exfoliated epithelial cells. American journal of physiology. Gastrointestinal and liver physiology.

[22] Davidson LA (1998). Non-invasive detection of fecal protein kinase C betaII and zeta messenger RNA: putative biomarkers for colon cancer. Carcinogenesis.

[23] Schwartz S (2012). A metagenomic study of diet-dependent interaction between gut microbiota and host in infants reveals differences in immune response. Genome biology.

[24] Knight JM (2014). Non-invasive analysis of intestinal development in preterm and term infants using RNA-Sequencing. Scientific reports.

[25] Eisenberg, E. & Levanon, E. Y. Human housekeeping genes are compact. *Trends in Genetics* **19**, 362–365, 10.1016/S0168-9525(03)00140-9. 10.1016/S0168-9525(03)00140-9 12850439

[26] Palmer C, Diehn M, Alizadeh AA, Brown PO (2006). Cell-type specific gene expression profiles of leukocytes in human peripheral blood. BMC Genomics.

[27] Munoz J (2012). The Lgr5 intestinal stem cell signature: robust expression of proposed quiescent ‘+4’ cell markers. The EMBO journal.

[28] Li N (2014). Single-Cell Analysis of Proxy Reporter Allele-Marked Epithelial Cells Establishes Intestinal Stem Cell Hierarchy. Stem Cell Reports.

[29] Fevr T, Robine S, Louvard D, Huelsken J (2007). Wnt/beta-catenin is essential for intestinal homeostasis and maintenance of intestinal stem cells. Molecular and Cellular Biology.

[30] Grun D (2015). Single-cell messenger RNA sequencing reveals rare intestinal cell types. Nature.

[31] Rothenberg ME (2012). Identification of a cKit(+) colonic crypt base secretory cell that supports Lgr5(+) stem cells in mice. Gastroenterology.

[32] Dalerba P (2011). Single-cell dissection of transcriptional heterogeneity in human colon tumors. Nature biotechnology.

[33] Trzpis M, McLaughlin PMJ, de Leij L, Harmsen MC (2007). Epithelial Cell Adhesion Molecule: More than a Carcinoma Marker and Adhesion Molecule. The American journal of pathology.

[34] el Marjou F (2004). Tissue-specific and inducible Cre-mediated recombination in the gut epithelium. Genesis (New York, N.Y.: 2000).

[35] Chi J-T (2007). Gene Expression Programs of Human Smooth Muscle Cells: Tissue-Specific Differentiation and Prognostic Significance in Breast Cancers. PLOS Genetics.

[36] Mariadason JM (2005). Gene expression profiling of intestinal epithelial cell maturation along the crypt-villus axis. Gastroenterology.

[37] Georgijevic S (2007). Spatiotemporal expression of smooth muscle markers in developing zebrafish gut. Developmental dynamics: an official publication of the American Association of Anatomists.

[38] Khazen W (2005). Expression of macrophage-selective markers in human and rodent adipocytes. FEBS Letters.

[39] Witten DM, Tibshirani RJ (2009). Extensions of sparse canonical correlation analysis with applications to genomic data. Statistical applications in genetics and molecular biology.

[40] Narimatsu K (2015). Toll-like receptor (TLR) 2 agonists ameliorate indomethacin-induced murine ileitis by suppressing the TLR4 signaling. Journal of gastroenterology and hepatology.

[41] Watanabe T (2014). Anti-tumour necrosis factor agents reduce non-steroidal anti-inflammatory drug-induced small bowel injury in rheumatoid arthritis patients. Gut.

[42] Robinson MD, McCarthy DJ, Smyth G (2010). K. edgeR: a Bioconductor package for differential expression analysis of digital gene expression data. Bioinformatics (Oxford, England).

[43] Whitfield-Cargile CM (2016). The microbiota-derived metabolite indole decreases mucosal inflammation and injury in a murine model of NSAID enteropathy. Gut microbes.

[44] Ananthakrishnan AN (2012). Aspirin, nonsteroidal anti-inflammatory drug use, and risk for Crohn disease and ulcerative colitis: a cohort study. Annals of internal medicine.

[45] Imaeda H (2012). Terminal-restriction fragment length polymorphism (T-RFLP) analysis for changes in the gut microbiota profiles of indomethacin- and rebamipide-treated mice. Digestion.

[46] Fukumoto K (2011). Role of tumor necrosis factor-alpha in the pathogenesis of indomethacin-induced small intestinal injury in mice. International journal of molecular medicine.

[47] DiLauro S, Crum-Cianflone NF (2010). Ileitis: When It Is Not Crohn’s Disease. Current gastroenterology reports.

[48] Kwo PY, Tremaine WJ (1995). Nonsteroidal anti-inflammatory drug-induced enteropathy: case discussion and review of the literature. Mayo Clinic proceedings.

[49] Bjarnason I (1987). Nonsteroidal antiinflammatory drug-induced intestinal inflammation in humans. Gastroenterology.

[50] Bertrand V (1998). Increase in tumor necrosis factor-alpha production linked to the toxicity of indomethacin for the rat small intestine. British journal of pharmacology.

[51] Singh DP, Borse SP, Nivsarkar M (2016). Clinical importance of nonsteroidal anti-inflammatory drug enteropathy: the relevance of tumor necrosis factor as a promising target. Translational Research.

[52] Qualls JE, Kaplan AM, van Rooijen N, Cohen DA (2006). Suppression of experimental colitis by intestinal mononuclear phagocytes. Journal of leukocyte biology.

[53] Nadatani Y (2012). High mobility group box 1 promotes small intestinal damage induced by nonsteroidal anti-inflammatory drugs through Toll-like receptor 4. The American journal of pathology.

[54] Higashimori A (2016). Mechanisms of NLRP3 inflammasome activation and its role in NSAID-induced enteropathy. Mucosal immunology.

[55] Zeino Z, Sisson G, Bjarnason I (2010). Adverse effects of drugs on small intestine and colon. Best practice & research. Clinical gastroenterology.

[56] Jia Q (2008). Reduced colitis-associated colon cancer in Fat-1 (n-3 fatty acid desaturase) transgenic mice. Cancer research.

[57] Davidson LA, Lupton JR, Miskovsky E, Fields AP, Chapkin RS (2003). Quantification of human intestinal gene expression profiles using exfoliated colonocytes: a pilot study. Biomarkers: biochemical indicators of exposure, response, and susceptibility to chemicals.

[58] Dobin A (2013). STAR: ultrafast universal RNA-seq aligner. Bioinformatics (Oxford, England).

[59] Zhang ZH (2014). A Comparative Study of Techniques for Differential Expression Analysis on RNA-Seq Data. PloS one.

[60] McQueen, C. M. *et al*. TRPM2 SNP genotype previously associated with susceptibility to Rhodococcus equi pneumonia in Quarter Horse foals displays differential gene expression identified using RNA-Seq. *BMC Genomics* **17**, 10.1186/s12864-016-3345-3 (2016). 10.1186/s12864-016-3345-3 PMC5139010 27919223

[61] McCarthy DJ, Chen Y, Smyth GK (2012). Differential expression analysis of multifactor RNA-Seq experiments with respect to biological variation. Nucleic acids research.

[62] Witten DM, Tibshirani R, Hastie T (2009). A penalized matrix decomposition, with applications to sparse principal components and canonical correlation analysis. Biostatistics (Oxford, England).

[63] Rouse M, Singh NP, Nagarkatti PS, Nagarkatti M (2013). Indoles mitigate the development of experimental autoimmune encephalomyelitis by induction of reciprocal differentiation of regulatory T cells and Th17 cells. British journal of pharmacology.

[64] Warde-Farley D (2010). The GeneMANIA prediction server: biological network integration for gene prioritization and predicting gene function. Nucleic acids research.

[65] Braga-Neto U, Dougherty E (2004). Bolstered error estimation. Pattern Recognition.

