# Supplementary material for: The non-invasive exfoliated transcriptome (exfoliome) reflects the tissue-level transcriptome in a mouse model of NSAID enteropathy
Source: Sci Rep. 2017 Oct 31;7:14687. doi: 10.1038/s41598-017-13999-5 (PMC5665873; doi:10.1038/s41598-017-13999-5)
Supplement: Supplementary file 1 — Supplementary Information [file 41598_2017_13999_MOESM1_ESM.pdf]

The non-invasive exfoliated transcriptome (exfoliome) reflects the tissue-level transcriptome in a mouse model of NSAID enteropathy

Authors: Canaan M. Whitfield-Cargile[1], Noah D. Cohen[1,5], Kejun He[2], Ivan Ivanov[3,5], Jennifer S. Goldsby[4,5], Ana Chamoun-Emanuelli[1], Brad R. Weeks[6], Laurie A. Davidson[4,5], Robert S. Chapkin[4,5]

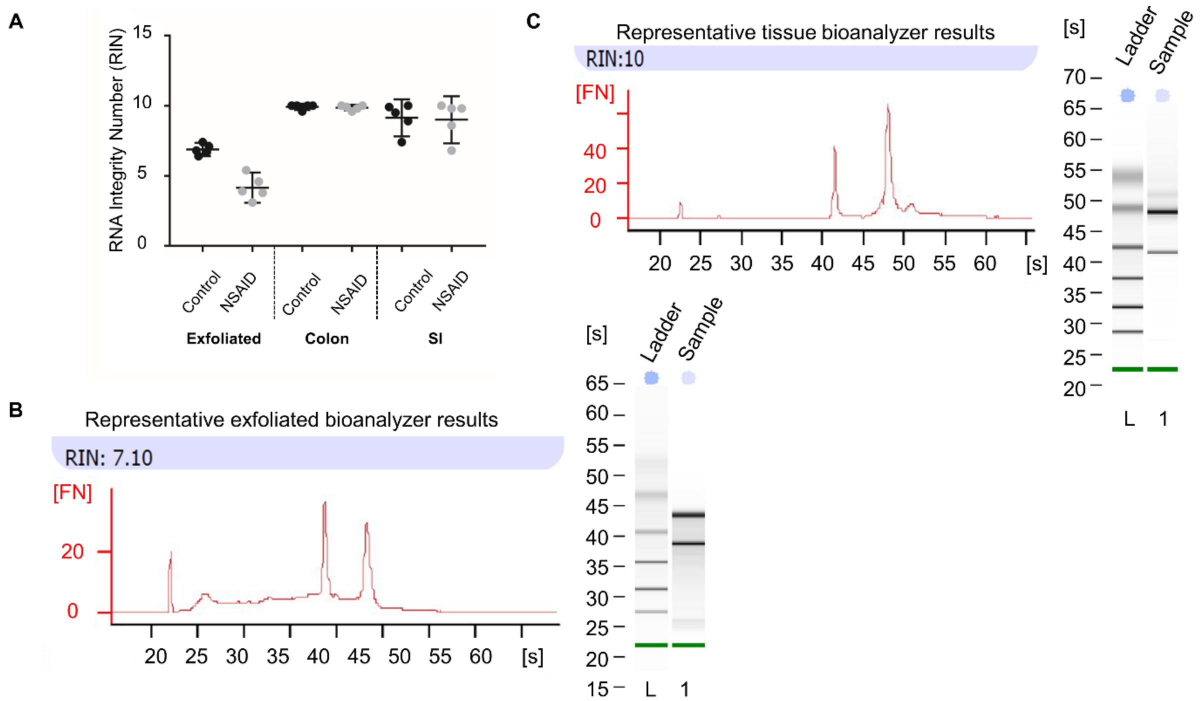

Supplementary Figure 1: Bioanalyzer results revealed lower quality total RNA in the exfoliome relative to the tissue due to partial RNAdegradation. **a –** Boxplots of RNA integrity number (RIN) from each dataset and treatment group. **b –** Representative Bioanalyzer results and virtual gel from exfoliated cell RNA. Note the presence of bacterial 23S and 16S ribosomal RNA. **c -** Representative Bioanalyzer results and virtual gel from tissue RNA (there were no differences between SI and colonic RNA). Note the presence of eukaryotic 28S and 18S ribosomal RNA.

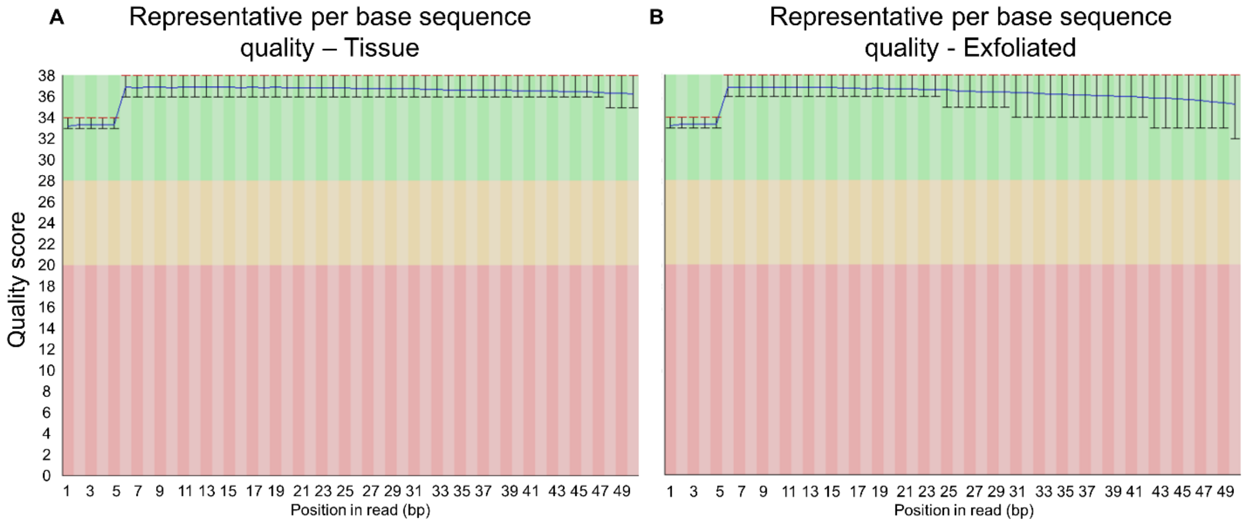

Supplementary Figure 2: FastQC results reveal similar per base sequence quality between exfoliated cell RNA and tissue RNA after sequencing. Representative per base quality scores for (**a**) exfoliated cell RNA and (**b**) colonic and SI tissue RNA after sequencing.

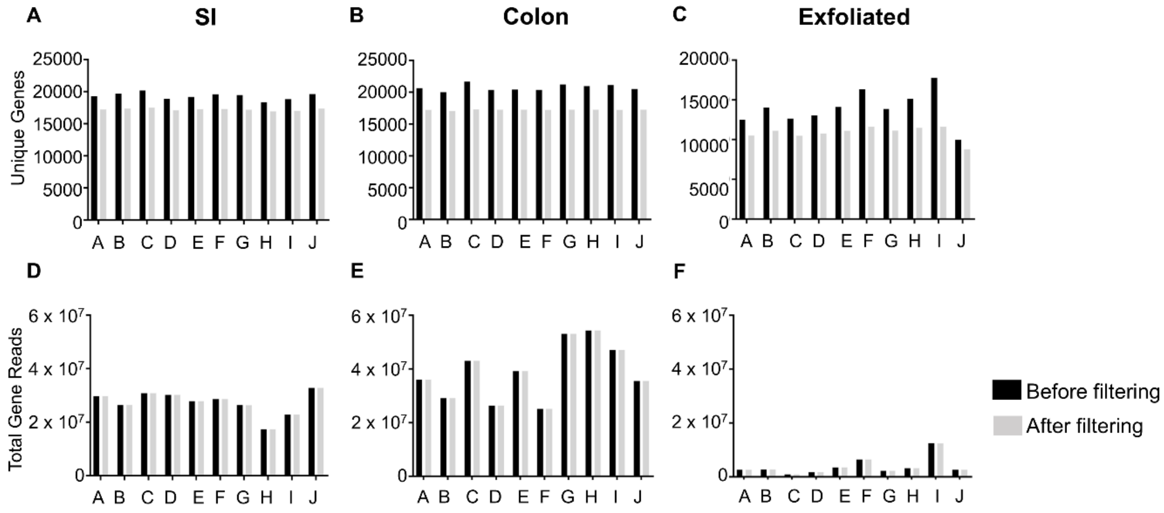

Supplementary Figure 3: Removing genes present in low abundance has minimal effect on numbers of unique genes or total gene reads in each sample across all treatment groups. Total number of unique genes present in each sample from the (**a**) SI transcriptome, (**b**) colonic transcriptome or (**c**) exfoliome before (black) and after (gray) removal of genes present in low abundance. Total number of reads present in each sample from the (**d**) SI transcriptome, (**e**) colonic transcriptome and (**f**) exfoliome before and after removal of genes present in low abundance.

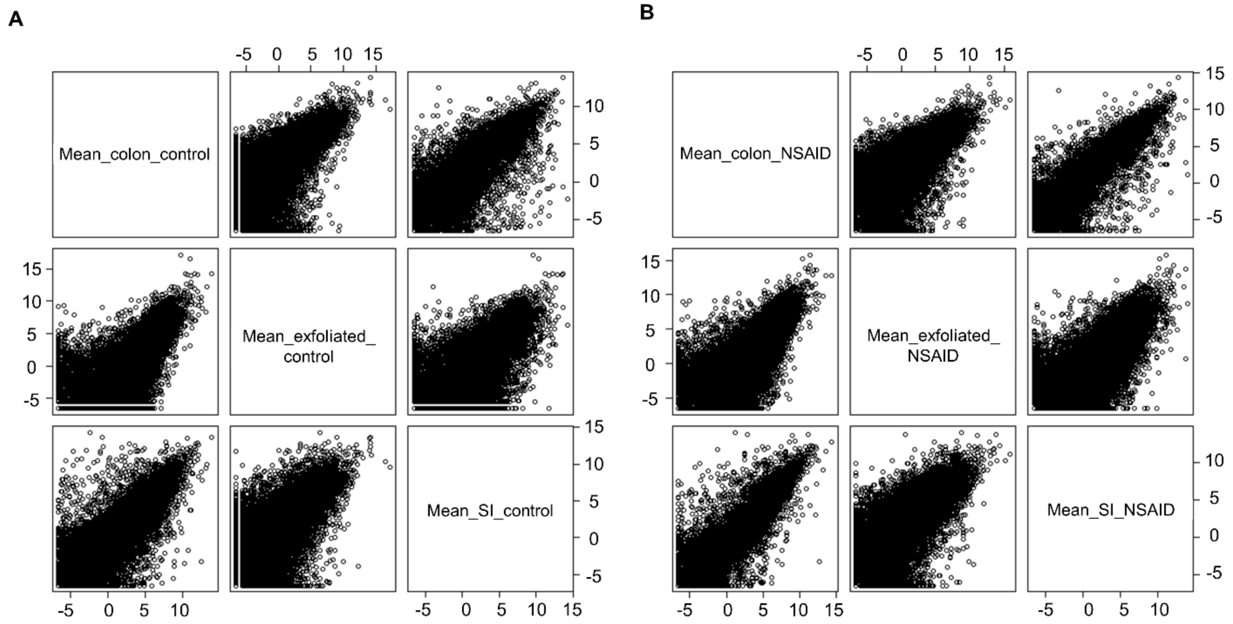

Supplementary Figure 4: Pairwise scatter plots of log(2) counts per million (CPM) for each gene in each treatment group (*i.e.*, NSAID and control) reveal correlation between all 3 datasets. Pairwise log(2) scatterplots between all 3 sources of data (i.e., colon, exfoliated, and SI) where the average log(2) expression is plotted for each source within the (**a**) control groups and (**b**) NSAID group.

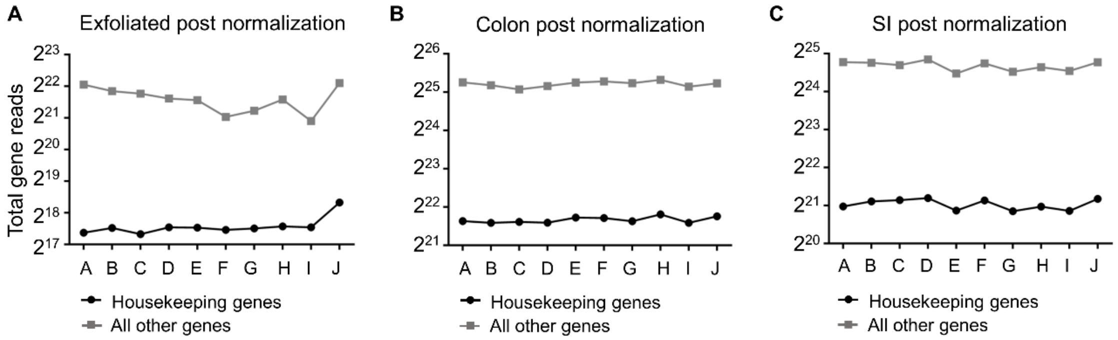

Supplementary Figure 5: log(2) total gene reads of 532 murine house-keeping genes (black) plotted against all other genes (grey) per sample for exfoliated cells (**a**), colonic mucosa (**b**), and SI mucosa (**c**) reveals analytical procedures effectively normalized for differences in library sizes.

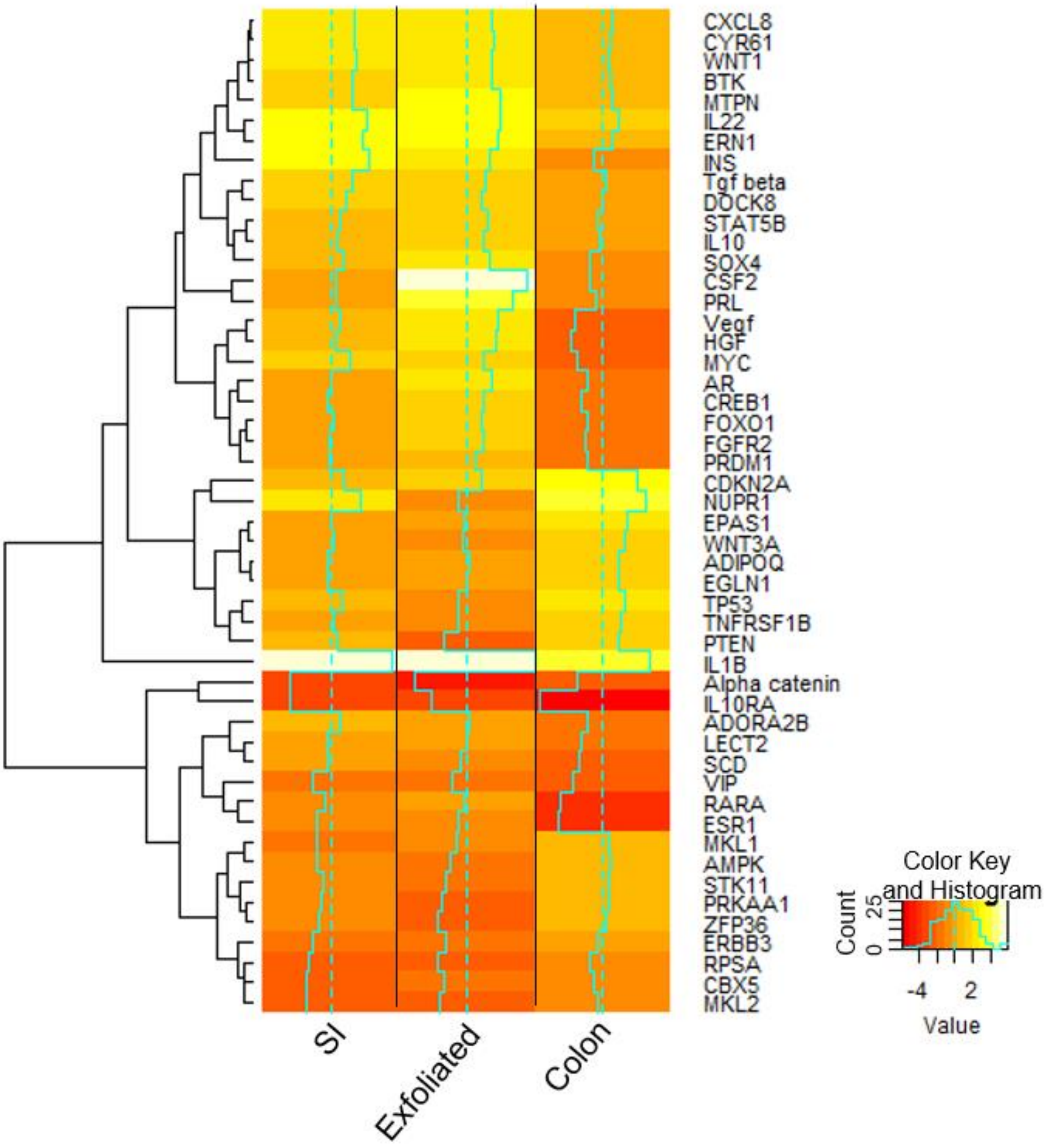

Supplementary Figure 6: Heatmap of genes identified as important upstream regulators of the canonical pathways shown in Figure 7. These data support the high degree of concordance between the SI transcriptome and exfoliome as compared with the colonic transcriptome.

Cell markers	Gene name
Crypt base columnar (CBC) cells	Ascl2
	Lgr5
	Nr2e3
	Tnfrsf19
Intestinal stem cell - CBC cells restricted	Aqp4
	Ascl2
	Cd44
	Cdca7
	Cdk6
	Clca4
	Kcnq1
	Msi1
	Nav1
	Smoc2
	Soat1
Intestinal stem cell signature – Within the crypt with highest expression at the crypt bottom	Afap1l1
	Agr3
	Cnn3
	Dach1
	Slc12a2
	Slco3a1
	Sorbs2
	Tns3
	Vdr
Quiescent/+4 stem cell markers expressed in CBC cells	Bmi1
	Hopx
	Lrig1
	Tert
Transit Amplifying (TA) cells	Axin2
	Ccnd1
	Cd44
	Myc
Absorptive enterocytes	Alpi
Hormone secreting enteroendocrine	Cck
	Chga
	Chgb
	Reg4
	Tac1
	Tph1

Tuft cells	Cd24a
	Dclk1
	Krt8
	Krt18
Goblet cells	Agr2
	Clca3
	Dll1
	Dll4
	Kit
	Muc2
Intestinal epithelail cells	Vil1
	DSC2
	Rab13
	Tfr
	Vil2
	Epcam
Neutrophils	Aqp9
	sod2
	il8RB/CXCR2
	PBEF1
	ly6g/Gr-1
	CD20/Ms4a1
B-Lymphocytes	B220/PTPRC
	CD74/HLA-DMA
	CEBPB
	a-sma
Smooth Muscle	HOXa10
	HNMT
	sm22/tagln
	f4/80 (EMr1)
Macrophages	cd14
	cd68
	CD86
	Defa5
Paneth cells	Ang4
	Nupr1
	Gfi1b

Table S1: A list of the genes used as biomarkers for each cell type.

Exfoliated	SI	Colon
4933431E20Rik	ADPRM	2010016I18Rik
ABCG5	ANKRD37	AGBL2
Akap9	ARF3	AZGP1
APAF1	ATP8B1	B230206H07Rik
APOBEC3B	BCL2L15	C5
ARHGDIB	CRYM	Celf6
ARNTL	CTDSP1	CLPS
ATG10	DOK4	ENC1
BTG1	Fam187b	KCTD12
CDK8	GCSAM	KPNA2
DEK	HM13	NDRG1
DHX32	KCTD5	OLR1
Dst	KRBA1	SLC2A10
EPS8L3	LRRC75A	TPPP
ESYT2	LXN	TRAF1
ETS2	NPTX1	WLS
GLTSCR1L	NR1D1	GM28373
GNA13	PLD2	
GRPEL2	PPP2CB	
HLA-A	PRUNE1	
IL15	Qars	
INTS6	RAPGEFL1	
KBTBD2	REG4	
KIN	SDR42E1	
LHFPL2	SLC16A11	
LPP	TBC1D10B	
MBNL2	TRPM2	
MCEMP1	TTC22	
MGEA5	VGLL4	
MYSM1	VNN1	
NHLRC2	ZDHHC24	
NUAK2	Rpl31-ps8	
PAPOLA	GM14480	
PMP22	GM9917	
PNRC1		
PPP1R12A		
PPP2R2A		
PRPF39		
RIN2		
SERINC2		
SLC34A2		
SLC35F5		

SPTBN1		
TNFAIP3		
TNFRSF11A		
TNFRSF21		
TOR1AIP2		
TRAPPC8		
Rasgef1b		

Table S2: Genes identified by LDA as being able to classify NSAID treated animals from control animals with less than 5% error.
